# Three-Month Treatment with Monoclonal Antibodies Targeting the CGRP Pathway Is Associated with Multi-Domain Changes in Sensory Processing and Cortical Network Efficiency in Migraine: Results from a Prospective Case–Control Study

**DOI:** 10.3390/biomedicines14050996

**Published:** 2026-04-27

**Authors:** Lara Klehr, Anne Thiele, Merle Bendig, Christine Kloetzer, Thorsten Herr, Nursena Armagan, Sebastian Strauss, Robert Fleischmann

**Affiliations:** 1 Department of Neurology, University Medicine Greifswald, 17475 Greifswald, Germany; lara.klehr@ukbonn.de (L.K.); christine.kloetzer@med.uni-greifswald.de (C.K.); thorsten.herr@med.uni-greifswald.de (T.H.); nursena.armagan@yahoo.com (N.A.); sebastian.strauss@med.uni-greifswald.de (S.S.); 2 Department of Neurology, University Hospital Bonn, 53127 Bonn, Germany

**Keywords:** migraine, headache, treatment, CGRP, monoclonal antibodies, sensory processing, electroencephalography

## Abstract

**Background/Objectives:** Monoclonal antibodies targeting the calcitonin gene-related peptide (CGRP) pathway are effective drugs for migraine prevention. The worsening of symptoms after treatment discontinuation has raised the question of whether these agents are associated with sustained central neurophysiological adaptation. This study investigated treatment-associated changes in sensory processing and cortical network efficiency during preventive treatment with CGRP monoclonal antibodies (mAbs). **Methods:** Twenty-two patients with episodic migraine (21 female, 46.2 ± 13.8 years) and 22 age- and sex-matched healthy controls underwent visual and somatosensory evoked-potential (VEP, SSEP) assessments and quantitative electroencephalography (qEEG). Patients were investigated before treatment initiation (V0) and after 3 months of CGRP mAb treatment (V3). Healthy controls were assessed once. **Results:** The lack of habituation of VEPs at V0 shifted toward habituation at V3 following treatment with CGRP mAbs (Δslope: −0.37 ± 0.83, *p* = 0.03). VEP habituation at V3 no longer differed significantly from controls. SSEP amplitudes remained stable and did not differ significantly between groups across the study interval. Exploratory qEEG parameters indicated a less efficient cortical network organization at V0 that was no longer significantly different from controls at V3. **Conclusions:** Three months of CGRP mAb treatment was associated with a partial normalization of selected neurophysiological parameters, particularly VEP habituation and exploratory qEEG network measures. Given the study design and small sample size, these findings indicate adaptive changes in multi-domain processing, yet these should not be overinterpreted as proof of disease modification.

## 1. Introduction

Migraine is a cycling disorder of sensory processing with a substantial medical and socioeconomic burden for affected individuals and society [1]. The availability of monoclonal antibodies (mAbs) targeting the calcitonin gene-related peptide (CGRP) pathway has ushered in a novel era of well-tolerated and effective preventive treatments [2]. However, their presumed peripheral mechanism of action, i.e., primarily targeting the trigeminovascular pathway, raises questions about their central disease-modifying capabilities and sustained therapeutic effects following treatment discontinuation [3,4]. Although the discontinuation of preventive treatment with CGRP mAbs is recommended in several national guidelines, the rapid deterioration of headache frequency and burden in the vast majority of patients after termination was repeatedly shown [5]. Neurophysiological and neuroimaging studies have suggested the improvement of central migraine-related measures during treatment, including hypothalamic activity and the dysfunctional brainstem processing of noxious stimuli [6,7]. These observations are intriguing, but they do not necessarily by themselves demonstrate a disease-modifying effect. Given that migraine is widely considered a disorder of multimodal sensory processing, one relevant question is whether preventive treatment with CGRP mAbs is associated with changes in higher-order sensory processing and cortical network organization beyond structures more directly linked to attack generation [8].

This study thus aims to clarify whether multi-domain sensory processing and cortical network efficiency are affected by preventive treatment with CGRP mAbs. Co-primary endpoints were interictal changes in electrophysiological measures of somatosensory and visual processing in patients affected by episodic migraine, which have frequently been reported as deficient in migraine [9], though not consistently across studies and with considerable interindividual variability [10,11,12]. Further endpoints involve the comparison of sensory processing between individuals with migraine and healthy controls as well as changes in global cortical network efficiency following three-month preventive treatment with CGRP mAbs [13].

## 2. Materials and Methods

### 2.1. Ethical Approval and Study Registration

This study was prospectively registered at clinicaltrials.gov (registration number: NCT04019496; date of registration: 15 July 2019) and approved by the local ethics committee (Ethikkommission an der Universitätsmedizin Greifswald, Germany; identifier: BB 168/18; date of approval: 13 November 2018). All procedures adhered to the Helsinki declaration in its latest revision and were conducted in line with current guidelines for good clinical practice (ICH E6(R2)). All patients and controls were provided detailed study information, and informed consent was obtained from all subjects prior to any study-specific procedures.

### 2.2. Study Design and Participant Selection

We enrolled 22 patients suffering from episodic migraine and 22 age-/gender-matched controls in this prospective case–control study. Patients with episodic migraine were recruited through the outpatient headache service of a tertiary care university hospital. All patients included in the study had to meet three distinct criteria. The episodic migraine had to be diagnosed using the ICHD-3. Furthermore, they needed to fulfill the requirement of failed treatment with four out of five preventive drugs for migraine, such as ß-Blockers, Flunarizine, Amitriptyline, Topiramate and Valproate. Finally, the completion of a three-month migraine diary was mandatory for acceptance into the study. Controls were selected from an internal database maintained by the neurology department of the University Hospital in Greifswald, which comprises individuals such as students, hospital staff, and other volunteers who have previously participated in studies and expressed interest in future research participation. To ensure a gender- and age-matched control group, we additionally recruited students to reflect the age distribution of the younger migraine participants.

Participants were assessed around the same time of the day by the same study personnel. The patients with migraine were tested twice during the study period. It is well established that neurophysiological properties can vary in close temporal proximity to migraine attacks, with changes occurring during the premonitory and postdromal phases. These fluctuations are attack-specific and may not reflect enduring changes in brain function. While some neurophysiological measures may remain stable during attacks, the interictal phase is particularly relevant when assessing treatment-associated changes in sensory processing and brain network activity. Our study therefore focused on capturing neurophysiological properties during the interictal interval. Patients were advised not to present for assessments when they had experienced a migraine attack within the preceding 24 h in order to ensure that data were collected during the interictal period. Patients with chronic migraine were excluded for the same reason. Baseline assessments were conducted before starting treatment with CGRP mAbs and included visual and somatosensory evoked potentials, as well as a 10 min resting EEG recording. This first visit was marked as V0 and ended with the first injection of a CGRP mAb. Three months later, patients repeated the neurophysiological assessments at V3. Healthy controls were investigated only once. This asymmetry in study design constitutes an important limitation for the interpretation of longitudinal changes in VEP habituation and EEG network parameters, because repeated testing, attentional state, and adaptation to the experimental environment may also contribute to within-patient change over time. In addition, exploratory chart-review data on monthly headache days at 6 and 12 months were available only for a subset of patients and were interpreted as descriptive, available-case follow-up information.

### 2.3. Primary and Secondary Endpoints

Changes in two domains of sensory processing following the three-month preventive treatment with CGRP mAbs in episodic patients with migraine were investigated as co-primary endpoints in this study. We used two different types of stimuli to investigate the multimodal sensory processing.

Visual evoked potentials (VEPs) were measured using pattern-reversal stimulation. Only one eye was tested per participant. In patients with strictly unilateral migraine, the eye ipsilateral to the headache side was examined; in patients with alternating-sided migraine, the eye on the side with the higher headache frequency was selected. Participants were seated 1.5 m in front of a screen displaying a checkerboard pattern of black and white squares (alternating at 3 Hz, luminance 50 cd/m^2^, contrast 80%, check size approximately 1 degree of visual angle) in a quiet, dimly lit room. Electrodes were placed in the midline over the occipital region 2.5 cm above the inion (Oz) as an active electrode and over the frontal region (Fz) as a reference. Signals were band-pass-filtered from 1 to 100 Hz. The ground electrode was placed on the arm. Data were sampled at 4 kHz. A total of 600 monocular stimuli were recorded per participant. VEP habituation was quantified by the standardized slope (beta coefficient) of the linear regression of VEP amplitudes (peak-to-peak N75–P100) against stimulation order, with values < 0 indicating habituation and values > 0 indicating a lack of habituation of successive responses. All trials were averaged offline in MATLAB R2025b (The MathWorks, Inc., Natick, MA, USA) across six consecutive blocks of 100 stimuli, and the habituation slope was derived from the block-wise amplitudes.

The somatosensory evoked potentials were elicited through electrical stimulation of the median nerve at the wrist. To reduce patient burden and fatiguing effects from extensive investigations, we combined the SSEP analyses with the EEG analyses. For this reason, electrodes for recording SSEPs were included in a 64-channel dry EEG system (see specifics of this technology in the subsection below) and recorded in its native equidistant layout. Cortical SSEP analysis was performed after offline remapping/interpolation to standard 64-channel electrode positions. According to the ACNS guidelines, CP3/CP4 denote the centroparietal positions halfway between C3/C4 and P3/P4 [14], and CP3/CP4 are operationalized as electrodes placed 2 cm posterior to C3/C4 [15]. Cortical responses were hence analyzed at CP3 or CP4 contralateral to the stimulated side, i.e., over the sensorimotor cortex, with mastoid reference as required in the ANT setup. Previous work has also shown that dry EEG provides evoked-potential and topographic information comparable to gel-based recordings, supporting such remapped analysis of evoked responses [16]. The ground electrode was placed on the arm. Each side was stimulated twice with 500 single stimuli, with a 1 min break between runs. Averaged traces were calculated offline for each run and each side and then averaged across sides to obtain a single SSEP waveform per participant. The stimulation frequency was 4.4 Hz, the sampling frequency was 2 kHz, and signals were band-pass-filtered from 30 to 1000 Hz. The intensity was set at 1.5 times the motor threshold. SSEP amplitude was defined as the baseline-to-peak amplitude of the N20 component. Habituation was quantified in analogy to VEPs as the standardized slope (beta coefficient) of the linear regression of block-wise amplitudes across stimulation order.

Secondary endpoints included comparisons of VEP and SSEP processing in patients with migraine at baseline and following three-month treatment with CGRP mAbs in comparison to healthy controls.

### 2.4. Exploratory Endpoints

Patients with migraine were shown to be characterized by changes in cortical network efficiency, both in electroencephalographic (EEG) and evoked-potential studies [13,17]. We therefore sought to investigate well-established measures of cortical network physiology using graph theory based on EEG data [18]. Resting-state EEG data was acquired using a 64-channel dry EEG channel system (Waveguard touch, ANT Neuro, Enschede, The Netherlands). Ten-minutes of EEG data were recorded with eyes closed and subsequently processed in a Matlab environment, which included band-pass filtering (0.5–100 Hz), notch filter (50 Hz) and the manual rejection of artifacts. Remaining 10 s epochs were then split into samples of 4096 data points per channel and imported into BrainWave (version 0.9.151.7.2., University Medical Center Amsterdam, Amsterdam, The Netherlands [19]). Brainwave yields sensor-level graph characteristics of cortical network efficiency expressed as minimum spanning tree metrics, computed separately for the delta (0.5–3 Hz), theta (4–7 Hz), and alpha (8–13 Hz) frequency bands. Kappa is an indicator of degree distribution, and larger values mean that network nodes are more distinctly weighted, which is considered more efficient [20]. The leaf fraction is the ratio of the number of nodes that have only one edge, and a higher leaf fraction indicates better network integration. The network diameter gives back the distance between any two nodes in the network, i.e., larger values are associated with poorer network integration. Tree hierarchy is evaluated as the final parameter, which is an indicator of the balance between the number of leaves and betweenness centrality, i.e., larger values are considered more beneficial since network efficiency increases while avoiding hub overloads.

### 2.5. Statistics

Statistical analyses were carried out using the Statistical Package for the Social Sciences (SPSS v25.0, IBM, Armonk, NY, USA). Continuous data were analyzed for normal distribution using histogram plots before descriptive and inferential analyses. Unless stated otherwise, normal distribution was confirmed. Descriptive normally distributed data are presented as mean ± standard deviation; non-normally distributed data are presented as median and interquartile range (IQR). The co-primary planned analyses comprised within-patient comparisons of VEP and SSEP parameters between V0 and V3 as well as cross-sectional comparisons between patients and healthy controls. Paired t-tests were used for comparisons between patients at baseline and after three months of treatment, and unpaired t-tests were used for comparisons between patients and controls. Inflation of alpha error across the co-primary endpoints was addressed using Bonferroni correction. Analyses involving responder/non-responder stratification, resting-state connectivity, and longer-term clinical correlations were considered exploratory and were interpreted accordingly. For EEG network parameters, inferential statistics were first performed using a global analysis of variance with network parameters, subject type (cases vs. controls), and visit (baseline vs. three-month follow-up) as factors. Significant results in the global test were followed by Tukey post hoc testing.

## 3. Results

We enrolled 22 patients (21 female, mean age 46.2 ± 13.8 years; four migraines with aura [MwA]) and 22 matched controls (21 female, mean age 47.6 ± 14.9 years). There were no dropouts in either group. Twelve patients received erenumab, five galcanezumab, and five fremanezumab for episodic migraine. Population characteristics are summarized in Table 1. Although these agents all target the CGRP pathway, they differ in their molecular target and dosing characteristics and thus introduce treatment heterogeneity that should be considered when interpreting the results. Headache diaries confirmed that all patients were assessed at least 24 h after their last migraine attack (distribution illustrated in Supplemental Figure S1), which in this cohort was sufficient to avoid statistically significant carry-over effects from temporally close premonitory or postdromal changes (see Supplemental Table S1). At baseline, the median headache frequency was 13 (IQR 10.5–14) days/month, the mean attack duration was 8.0 ± 5.3 h/attack, and the mean headache intensity was 4.9 ± 1.9. After 3 months of CGRP mAb treatment, headache frequency [5 (IQR 3–10) days/month, *p* < 0.001 vs. V0] and attack duration (5.4 ± 4.2 h/attack, *p* = 0.014 vs. V0) decreased significantly, whereas headache intensity remained unchanged (5.1 ± 2.5, *p* = 0.96 vs. V0). Twelve patients achieved a reduction in monthly headache days of ≥50% after 3 months and were classified as responders. Exploratory available-case chart-review data suggested that monthly headache days remained lower at 6 months and 12 months (5.7 ± 4.1 days/month and 4.9 ± 3.3 days/month, respectively) (see Supplemental Table S2). Yet these later observations were not protocolized study assessments.

### 3.1. Visual Evoked Potentials

**Figure 1:**
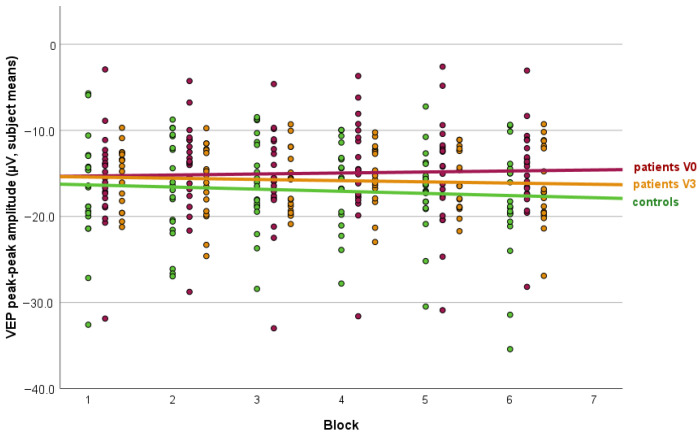
VEP amplitudes in controls and migraine patients before and during CGRP monoclonal antibody treatment. The figure displays the average peak-to-peak amplitudes of visual evoked potentials (VEPs) per stimulus block across three conditions: healthy controls, migraine patients before treatment, and migraine patients three months after initiation of CGRP mAb therapy. Fit lines are shown for each group to illustrate the amplitude trends across blocks. While overall amplitude levels are comparable between groups, the migraine group at baseline exhibits a clear pattern of lack of habituation over successive blocks, in contrast to the habituation observed in healthy controls and in patients following treatment. These trends indicate that habituation deficits at baseline are normalized after three months of preventive CGRP-targeted therapy. Stacked colored dots in each column correspond to patients, controls, and visits, and use the same color coding as the corresponding regression lines.

The mean VEP amplitudes across stimulation blocks were significantly lower in patients at baseline (14.9 ± 5.5 µV) than in controls (16.9 ± 5.6 µV, *p* = 0.006). A repeated-measures ANOVA in the patient group revealed a significant change in VEP amplitudes across visits following three months of treatment with CGRP monoclonal antibodies (F(17, 85) = 114.4, *p* < 0.001), whereas no significant block-wise effect was observed (F(85, 202) = 0.9, *p* = 0.69). After three months, VEP amplitudes in patients increased to 15.8 ± 3.8 µV and no longer differed significantly from controls (*p* = 0.074). The slope parameter of VEP habituation differed significantly between patients with migraine and controls, indicating a lack of habituation in the migraine group (0.25 ± 0.57 [range: −0.78 to 1.36]) and habituation in controls (−0.20 ± 0.89 [range: −1.85 to 1.34], *p* = 0.031). After three months of treatment, VEP habituation in the migraine group shifted toward habituation (−0.12 ± 0.85 [range: −1.52 to 2.08], *p* = 0.03 vs. V0) and no longer differed significantly from controls (*p* = 0.40). The results are summarized in Figure 1.

### 3.2. Somatosensory Evoked Potentials

SSEP amplitudes remained stable across the treatment interval in migraine patients and did not differ significantly from controls at either baseline or follow-up. In patients, the mean baseline-to-peak N20 amplitude was 0.641 ± 0.239 µV at V0 and 0.652 ± 0.246 µV at V3. This numerical increase was not statistically significant (*p* = 0.36). In between-group comparisons, SSEP amplitudes in patients did not differ from healthy controls at baseline or after treatment. There was also no significant difference in SSEP habituation between patients with migraine and controls at baseline, with findings indicating habituation in both groups (migraine: −1.13 ± 0.16 [range: −1.48 to −0.86], controls: −1.12 ± 0.15 [range: −1.44 to −0.86], *p* = 0.43). SSEP habituation in patients at three-month follow-up (−1.09 ± 0.12 [range: −1.34 to −0.91]) was unchanged compared with baseline (*p* = 0.19) and remained not significantly different from controls (*p* = 0.41).

### 3.3. Resting-State Connectivity

**Figure 2:**
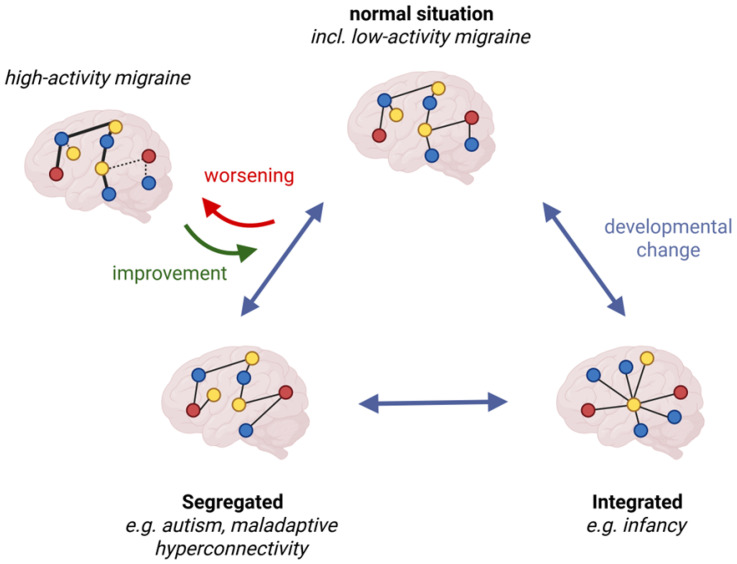
Schematic representation of sensor-level connectivity changes in migraine with and without preventive treatment. Simplified brain network organizations are depicted across developmental stages, including how findings in migraine patients align with graph–theoretical network models. In patients who did not receive preventive treatment, the network diameter was larger, and Kappa values were lower, indicating a more segregated and less resilient network architecture. After three months of treatment with CGRP mAbs, network parameters became indistinguishable from those of matched control subjects. Different node colors are used arbitrarily and solely for illustrative purposes to depict hubs across different networks.

EEG analyses were exploratory in nature, and an overview of the descriptive data of all network parameters across frequency bands can be found in Table 2, Table 3 and Table 4. Minimum spanning tree parameters assessed at V0 significantly differed between the migraine group and controls regarding Kappa (F = 5.6, *p* = 0.02), which was not frequency-specific (migraine: 6.6 ± 2.7, controls: 7.2 ± 2.6). There was furthermore a significant frequency-specific difference regarding the diameter at baseline (F = 6.5, *p* = 0.002). Post hoc testing revealed that the diameter in the migraine group was larger in the alpha band (migraine: 0.56 ± 0.14, controls: 0.46 ± 0.10, *p* = 0.03). Kappa (F = 2.89, *p* = 0.15) and diameter (F = 1.60, *p* = 0.21) assessed in patients with migraine were not significantly different from control values after three months of treatment (schematic of connectivity changes in Figure 2). Neither the tree hierarchy nor the leaf fraction significantly differed between patients and controls at any time or frequency band (all *p* > 0.05).

### 3.4. Relationship Between Changes in Neurophysiological Parameters and Treatment Response

We explored whether changes in neurophysiological measures were related to clinical treatment response. Correlation analyses between changes in monthly headache days after 3 months of treatment and alterations in VEPs, SSEPs, and resting-state connectivity revealed no significant associations for the primary evoked-potential measures or the exploratory connectivity measures. We then performed binary comparisons between treatment responders and non-responders, defining responders as patients with a reduction of ≥50% in monthly headache days at 3 months. Again, connectivity measures showed no significant association with treatment response. Evoked-potential analyses showed different results. While VEP habituation did not significantly differ between responders and non-responders, the mean changes were 0.2 (95% CI, −0.4 to 0.7) in responders and −0.4 (95% CI, −1.7 to 0.8) in non-responders (*p* > 0.05). SSEP habituation likewise showed no significant group difference, with mean changes of −0.1 (95% CI, −0.2 to 0.1) in responders and 0.0 (95% CI, −0.2 to 0.1) in non-responders (*p* > 0.05). In contrast, responders showed a greater reduction in SSEP amplitudes, with a mean change of −0.01 µV (95% CI, −0.08 to 0.05) compared with 0.07 µV in non-responders (95% CI, −0.00 to 0.14; *p* = 0.032). Exploratory available-case analyses were also performed for later clinical course. Changes in evoked-potential measures at 3 months did not correlate significantly with later headache-frequency change (all *p* > 0.05).

In the exploratory connectivity analyses, a decrease in headache frequency was associated with an increase in theta-band leaf fraction (r = 0.42, *p* = 0.042). There were further non-significant trends for theta-band Kappa (r = −0.31, *p* = 0.093) and delta-band diameter (r = 0.32, *p* = 0.092).

## 4. Discussion

This study identified treatment-associated changes in sensory processing and brain network efficiency in a small cohort of patients with episodic migraine after three months of CGRP mAb treatment. In particular, the lack of habituation of VEPs at baseline shifted toward habituation during treatment, and exploratory EEG network measures were no longer significantly different from control values at follow-up. These findings are relevant because altered sensory processing and network organization have been implicated in migraine pathophysiology beyond structures directly involved in attack generation. At the same time, the interpretation of these longitudinal changes requires caution. Healthy controls were assessed only once, whereas patients were tested repeatedly at baseline and three months. Accordingly, the observed within-patient changes cannot be attributed with certainty to treatment alone and may also reflect repeated testing, attentional state, or adaptation to the experimental setting.

### 4.1. Changes in Evoked-Potential Studies in Relation to Previous Findings

Migraine is widely regarded as a disorder of altered sensory processing, and numerous studies have described the reduced habituation of evoked responses between attacks [8,21]. In the present study, we observed a lack of habituation of VEPs in the migraine group at baseline and a shift toward habituation after three months of treatment. This finding is broadly consistent with earlier reports, while also acknowledging that the literature is heterogeneous and that not all well-controlled studies have confirmed abnormal habituation in interictal migraine [10,12]. In addition, substantial interindividual variability has been reported even among healthy controls, which limits the usefulness of habituation measures as single-subject biomarkers [11]. Our own control group also showed a broad range of slope values, underscoring the need for cautious interpretation at the group level.

Several interventional studies have shown that preventive migraine treatments can alter the amplitudes and habituation of sensory evoked potentials. Changes in VEP and SSEP habituation have, for example, been described after the ketogenic diet and repetitive transcranial magnetic stimulation [22,23]. Against this background, our results do not imply a unique mechanism of CGRP monoclonal antibodies. Rather, they show that treatment with a CGRP mAb is likewise associated with measurable changes in cortical sensory physiology, most clearly in the visual domain.

In contrast to the VEP findings, the SSEP results were weaker. Baseline SSEP habituation did not differ significantly from controls, and treatment-related changes were limited. This differs from some prior migraine studies, including reports of altered SSEP habituation or high-frequency oscillations [24,25,26]. Several explanations are conceivable, including differences in patient selection, migraine cycle, baseline cortical state, and the generally greater variability of somatosensory evoked responses. Importantly, the present data suggest that the strongest treatment-associated signal in this cohort stems from visual processing rather than from a uniform multimodal normalization across all sensory modalities. This more restrained interpretation is, in our view, better aligned with the actual strength of the findings.

The interpretation of modality-specific findings is also supported by prior work linking VEPs to altered visual responsiveness in migraine and by studies suggesting that somatosensory evoked responses reflect altered somatosensory or pain-related cortical processing [27,28]. In this context, the VEP changes observed here may be more sensitive to treatment-associated modulation than the SSEP measures used in the present study.

### 4.2. Changes in Cortical Network Efficiency in Relation to Previous Findings

Beyond the sensory evoked-potential results, the present study also explored whether treatment with CGRP mAbs was associated with changes in resting-state cortical network organization. At baseline, the migraine group showed lower Kappa values and increased alpha-band diameter, findings that are compatible with a less integrated and potentially less efficient network architecture. After three months of treatment, these differences were no longer statistically significant when compared with controls. Because these EEG analyses were exploratory and because healthy controls were not reassessed longitudinally, these observations should be interpreted cautiously. Nevertheless, they are of interest because prior work has suggested altered large-scale network organization and altered sensory network integration in migraine [13,18,20].

Previous studies investigating the central effects of CGRP mAbs on EEG-derived measures have also suggested treatment-associated changes in cortical processing, although study designs, analytic approaches, and outcome measures differed from the present work [29,30]. Our findings are therefore best interpreted as convergent exploratory evidence rather than definitive proof of normalization of resting-state connectivity. It is also important to note that the absence of repeated control assessments prevents us from determining whether the observed within-patient changes reflect treatment-specific effects or, at least in part, retest-related or time-dependent influences.

Mechanistically, it is not expected that CGRP mAbs directly alter cortical information processing through major blood–brain barrier penetration [3]. A more plausible interpretation is that reduced peripheral trigeminovascular nociceptive input and reduced central sensitization secondarily modulate thalamocortical interactions, cortical responsivity, and large-scale network organization over time. Such indirect mechanisms remain hypothetical but are biologically plausible and may help explain why electrophysiological changes can accompany clinical improvement without constituting direct evidence of disease modification.

### 4.3. Neurophysiological Findings and Possible Clinical Implications

The question of whether CGRP-targeted therapy exerts disease-modifying effects in migraine remains open. On the one hand, several patients deteriorate again after the discontinuation of treatment, which argues against a robust and uniform disease-modifying effect in the strict sense. On the other hand, treatment-associated changes in central neurophysiological measures may still be relevant because they suggest that peripheral blockade of the CGRP pathway can be accompanied by secondary changes in cortical sensory processing and network organization.

In the present study, the most robust signal was the shift in VEP habituation toward control-like values. Exploratory EEG findings were directionally similar, but they were obtained in a more weakly powered analytic setting and therefore should be interpreted cautiously. The later chart-review data suggest that some patients maintain a lower headache frequency over longer intervals, but these observations were incomplete, non-protocolized, and cannot establish a mechanistic relationship between early neurophysiological change and longer-term clinical course. Accordingly, our data should not be read as evidence that CGRP mAbs modify the course of migraine. Rather, they suggest that treatment may be associated with the partial normalization of selected neurophysiological parameters in some patients.

This more cautious interpretation also aligns with the broader literature. Many preventive treatments have been reported to influence evoked-potential amplitudes and habituation, and it would therefore be inappropriate to regard the present findings as unique proof of disease modification by monoclonal antibodies. Future prospective studies with larger samples, repeated longitudinal control assessments, and ideally follow-up after treatment discontinuation are needed to determine whether early neurophysiological changes predict sustained clinical benefit and whether any subgroup-specific pattern of longer-lasting adaptation can be identified.

### 4.4. Limitations

Our findings should be interpreted in light of several limitations. First, the sample size was small, which limits precision, increases vulnerability to interindividual variability, and is particularly relevant for subgroup analyses such as responder/non-responder comparisons. Second, the treatment group was heterogeneous, as patients received three different CGRP monoclonal antibodies with different molecular targets and dosing characteristics. Third, healthy controls were assessed only once. This is a major limitation, because repeated testing, attentional state, and adaptation to the experimental setting may contribute to within-patient longitudinal change and therefore reduce causal certainty regarding treatment effects. Fourth, several analyses, particularly the EEG network analyses and later clinical correlations, were exploratory and should be interpreted as such. Fifth, we did not assess interictal symptoms or other clinical correlates of altered sensory processing directly; therefore, any link between neurophysiological change and the broader interictal migraine phenotype remains inferential. Finally, the later 6- and 12-month clinical data were based on available-case chart review rather than standardized protocol visits and therefore do not permit strong longitudinal conclusions.

We considered more complex statistical models to further investigate observed effects. Yet, given the modest sample size, the absence of repeated control assessments, and the exploratory nature of several analyses, this would inevitably lead to very limited interpretations. Future studies should be powered and designed to include more complex models to further enhance the understanding of observed effects, which cannot be delivered in the current design.

## 5. Conclusions

In this study, three months of treatment with CGRP monoclonal antibodies was associated with the partial normalization of selected neurophysiological measures in episodic migraine, most clearly in the visual domain and in exploratory qEEG network parameters. These findings support the view that peripheral CGRP pathway blockade may be accompanied by secondary central neurophysiological changes. However, given the small sample size, treatment heterogeneity, exploratory analyses, and absence of repeated control assessments, the data do not provide robust evidence for disease modification. Larger longitudinal studies with protocolized long-term follow-up and post-discontinuation assessment are needed to clarify the clinical significance of these findings.

## Figures and Tables

**Table 1:** Baseline demographic and clinical characteristics of the study population.

Variable	Migraine Patients (n = 22)	Healthy Controls (n = 22)
Age, years	46.2 ± 13.8	47.6 ± 14.9
Female sex, n (%)	21 (95.5)	21 (95.5)
Migraine with aura, n (%)	4 (18.2)	NA
Monthly headache days at baseline	13 (IQR 10.5–14)	NA
Attack duration at baseline, h/attack	8.0 ± 5.3	NA
Headache intensity at baseline	4.9 ± 1.9	NA
CGRP mAb treatment: erenumab/galcanezumab/fremanezumab (n)	12/5/5	NA

**Table 2:** MST parameters in the delta band.

		MST Kappa	MST Diameter	MST Leaf Fraction	MST Tree Hierarchy
**Baseline**					
Migraine	Mean	3.0221	0.2225	0.5311	0.3946
	Std. Deviation	0.34729	0.04694	0.04215	0.05318
Control	Mean	4.0794	0.2018	0.5983	0.4342
	Std. Deviation	1.51373	0.07323	0.07306	0.06388
**3-Month Follow-Up**					
Migraine	Mean	5.9637	0.1841	0.7079	0.4218
	Std. Deviation	3.41416	0.08403	0.18222	0.08640

**Table 3:** MST parameters in the theta band.

		MST Kappa	MST Diameter	MST Leaf Fraction	MST Tree Hierarchy
**Baseline**					
Migraine	Mean	8.3196	0.4008	0.4722	0.3634
	Std. Deviation	0.93896	0.11430	0.10758	0.09007
Control	Mean	8.4580	0.5000	0.4950	0.3679
	Std. Deviation	1.30315	0.08165	0.07619	0.04777
**3-Month Follow-Up**					
Migraine	Mean	9.8294	0.3774	0.6448	0.4197
	Std. Deviation	3.32601	0.16142	0.22045	0.07960

**Table 4:** MST parameters in the alpha band.

		MST Kappa	MST Diameter	MST Leaf Fraction	MST Tree Hierarchy
**Baseline**					
Migraine	Mean	8.5092	0.5588	0.5051	0.3381
	Std. Deviation	1.07409	0.13974	0.08995	0.09467
Control	Mean	9.0091	0.4600	0.4850	0.3340
	Std. Deviation	1.54446	0.10488	0.07835	0.06127
**3-Month Follow-Up**					
Migraine	Mean	8.5089	0.4522	0.5596	0.3702
	Std. Deviation	3.19379	0.17578	0.22516	0.12956

## Data Availability

The datasets generated and/or analyzed during the current study are not publicly available due to legal restrictions on data sharing under the EU General Data Protection Regulation (GDPR; in Germany: Datenschutz-Grundverordnung, DSGVO—Regulation (EU) 2016/679) but are available from the corresponding author on request.

## Supplementary Materials

The following supporting information can be downloaded at: https://www.mdpi.com/article/10.3390/biomedicines14050996/s1, Supplemental Figure S1: Occurrence of migraine attacks before and after measurement at baseline; Supplemental Table S1: Multivariate test of the statistical effect of assessment times to or from the next migraine attack on sensory processing; Supplemental Table S2: Number of available clinical follow-up observations at 6 and 12 months.
