# Supplementary material for: Three-Month Treatment with Monoclonal Antibodies Targeting the CGRP Pathway Is Associated with Multi-Domain Changes in Sensory Processing and Cortical Network Efficiency in Migraine: Results from a Prospective Case–Control Study
Source: Biomedicines. 2026 Apr 27;14(5):996. doi: 10.3390/biomedicines14050996 (PMC13204497; doi:10.3390/biomedicines14050996)
Supplement: Supplementary file 1 [file biomedicines-14-00996-s001.zip › biomedicines-4221203-supplementary.pdf]

Supplemental figure S1, occurrence of migraine attacks before and after measurement at baseline. There were no migraine attacks within 24 hours of assessments.

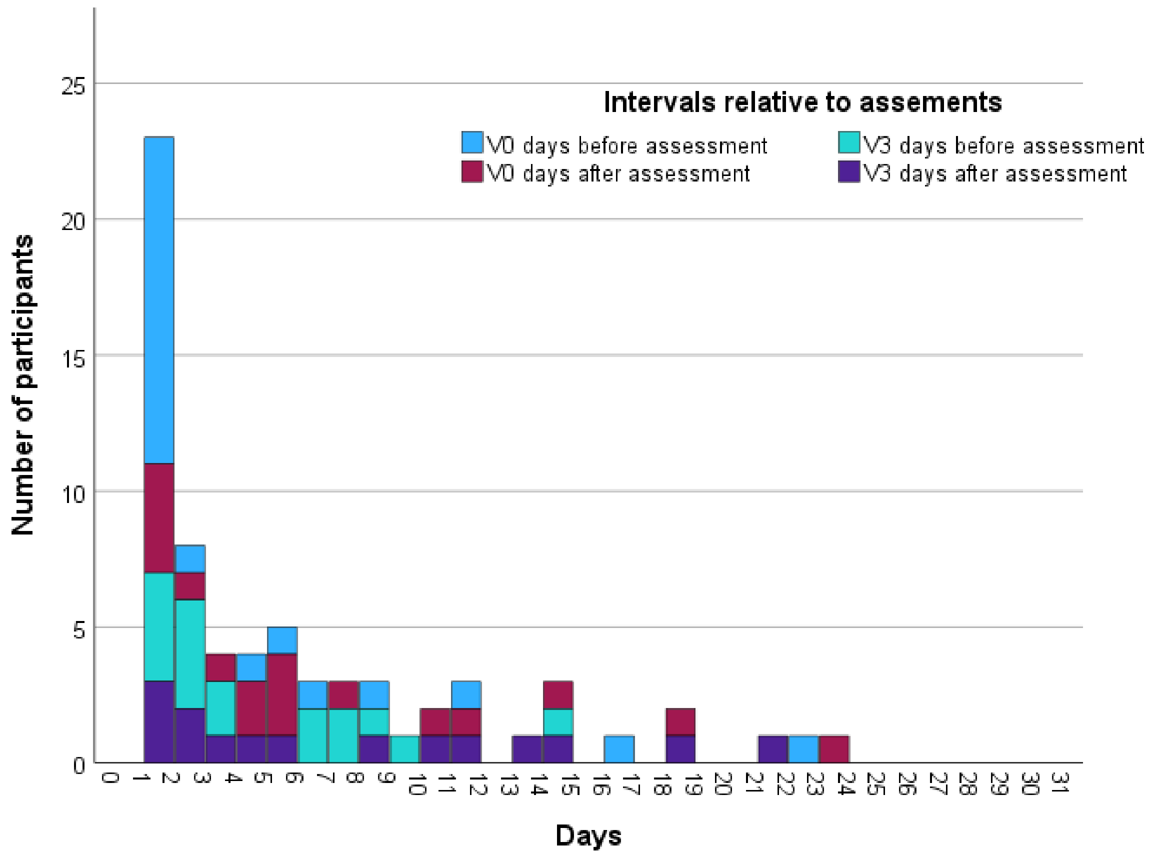

Supplemental table S1, multivariate test of the statistical effect of assessment times to or from the next migraine attack on sensory processing.

Effect	Value	F	Hypothesis df	Error df	Sig.
Intercept	.995	130.746[b]	6.000	4.000	<.001
ATKPREV0	.532	.758[b]	6.000	4.000	.638
ATKPOSV0	.482	.620[b]	6.000	4.000	.714
ATKPREV3	.427	.497[b]	6.000	4.000	.788
ATKPOSV3	.722	1.734[b]	6.000	4.000	.309

Design: Intercept + ATKPREV0 + ATKPOSV0 + ATKPREV3 + ATKPOSV3

ATKPREV0 = days migraine attack occurred before assessment at V0

ATKPOSV0 = days migraine attack occurred after assessment at V0

ATKPREV3 = days migraine attack occurred before assessment at V3

ATKPOSV3 = days migraine attack occurred after assessment at V3

Supplemental table S2, number of available clinical follow-up observations at 6 and 12 months.

Time point	Available observations, n
Neurophysiological assessment	22
Exploratory chart-review follow-up at 6 months	19
Exploratory chart-review follow-up at 12 months	18
